# Sex-specific Associations between Immune Parameters and Clinical Symptoms in First-episode Patients with Schizophrenia

**DOI:** 10.2174/011570159X354419250217100855

**Published:** 2025-03-10

**Authors:** Anle Pan, Meihong Xiu, Jiahong Liu, Jing Yao, Yuanyuan Huang

**Affiliations:** 1 The Affiliated Kangning Hospital of Wenzhou Medica University Zhejiang Provincial Clinical Research Center for Mental Disorder, Wenzhou, China;; 2 Peking University HuiLongGuan Clinical Medical School, Beijing HuiLongGuan Hospital, Beijing, China;; 3 Department of Psychiatry, The Affiliated Brain Hospital, Guangzhou Medical University, Guangzhou, China;; 4 Guangdong Engineering Technology Research Center for Translational Medicine of Mental Disorders, Guangzhou, China;; 5 Key Laboratory of Neurogenetics and Channelopathies of Guangdong Province and the Ministry of Education of China, Guangzhou Medical University, Guangzhou, China

**Keywords:** Schizophrenia, immune, neutrophil, lymphocyte, NLR, mental disorder

## Abstract

**Introduction:**

Inflammation is linked to the pathophysiology of schizophrenia. The neutrophil-to-lymphocyte ratio (NLR), a measure of systemic inflammation, has been reported to be associated with schizophrenia. However, few studies have examined the sex-specific association between neutrophil-to-lymphocyte ratio (NLR) and clinical symptoms in schizophrenia. This study aimed to explore sex differences in NLR and its correlation with symptoms in first-episode schizophrenia (FES) patients.

**Methods:**

Ninety-seven FES patients and 65 control subjects were recruited. The severity of clinical symptoms was assessed using the Positive and Negative Syndrome Scale (PANSS), and white blood cells were calculated. We performed a cross-sectional analysis comparing NLR in males and females in the patient and control groups. We explored its sex-specific associations with clinical symptoms in the patient group.

**Results:**

We found that neutrophil (NEU) counts and NLR were higher in male patients compared to female patients with schizophrenia. There were no significant differences in white blood cell counts and NLR in healthy controls. Linear regression analysis showed that NEU counts were associated with clinical symptoms in male patients, and NLR correlated with symptoms in female patients after controlling for age, onset age, and years of education.

**Conclusion:**

Our study suggests that NLR values and NEU counts were higher in male patients compared with female patients with schizophrenia and that the association between NLR or NEU and clinical symptoms was sex-specific.

## 1 INTRODUCTION

Schizophrenia is a severe mental disorder. There is growing evidence that patients with schizophrenia are associated with abnormal levels of immune system markers, including cytokines and immune cells, in postmortem studies and clinical studies [1-4]. In addition, anti-inflammatory agents as adjuvants to antipsychotics may alleviate clinical symptoms but produce without notable side effects [5, 6]. In particular, previous studies have demonstrated significant alterations in the secretion or production of cytokines from isolated blood monocytes in patients with schizophrenia compared to healthy controls [7, 8]. A subgroup of schizophrenia patients with high inflammatory components had increased expression of perivascular macrophage and astrocyte markers [9] and reduced concentrations of inhibitory interneuron markers in the brain [10].

White blood cells (WBC) play a crucial role in the peripheral immune system and the host immune response. Regarding immune cell components, most of the previous literature on schizophrenia has analyzed the mononuclear phagocyte system, such as peripheral blood mononuclear cells (PBMC) [11-13]. Little is known about the involvement of granulocytes (neutrophils, basophils, and eosinophils) in patients with schizophrenia [14], which account for approximately 50-80% of all WBC. *In vivo*, neutrophils (NEU) reflect non-specific inflammation, and lymphocytes (LYM) indicate specific immunity [15, 16]. Recently, significant changes in NEU and LYM counts have been observed in patients with schizophrenia and correlated with clinical symptoms [14, 17]. In particular, several recent meta-analyses have shown an increased circulating NEU to LYM ratio (NLR) in patients with schizophrenia [18, 19]. Thus, the imbalance between NEU and LYM counts in schizophrenia may be related to its pathophysiology.

Sex differences have been widely observed in different aspects of schizophrenia throughout the course of the illness [20-23]. However, there are no studies that have examined the impact of sex on NLR and its relationship with clinical symptoms. On the other hand, NLR, an inexpensive and reproducible biomarker of systemic inflammation, is a relatively new biomarker in the field of schizophrenia. Therefore, this study aimed to investigate 1) whether there were sex differences in NEU counts, LYM counts, and NLR values in patients with schizophrenia and healthy controls and 2) whether there were sex differences in the association between NEU, LYM, and NLR and clinical symptom severity.

## 2 METHODS

### 2.1 Patients

It is a cross-sectional study. Ninety-seven FES patients (male/female = 55/42) were recruited from four psychiatric hospitals in China. All participants were diagnosed with schizophrenia according to the Diagnostic and Statistical Manual of Mental Disorders, 4^th^ Edition (DSM-IV) criteria and confirmed by the Structured Clinical Interview for DSM-IV (SCID). The inclusion criteria were 1) Han nationality, 2) aged 16-45 years, 3) male or female, 4) first episode, 5) duration of illness < 60 months, and 6) normal laboratory and electrocardiographic measurement and written informed consent were also required at screening. None of the patients had received prior treatment with antipsychotics or had been on antipsychotics for less than two weeks. The exclusion criteria were: 1) substance dependence, 2) severe physical illness, 3) any comorbid central nervous system disorder, 4) meeting DSM-IV criteria for mental retardation, 5) use of anti-immune agents, and 6) inability to understand the study protocol.

Of the 97 patients in this study, 94 had a disease duration of less than 3 years. Additionally, 45 patients had previously been treated with antipsychotics. The duration of medication exposure was less than 6 days, and the mean treatment period with antipsychotics was 2.5 days.

We also enrolled sixty-five sex- and age-matched healthy control subjects during the period of patient enrolment. Healthy controls were excluded if they had a diagnosis of Axis I disorder or had a family history of psychiatric disorders using the SCID. The medical history and physical examination were obtained from each subject to exclude those with physical illness.

Patients and controls were excluded if they were taking anti-inflammatory drugs. Considering that acute infections could also affect WBC counts, we used CRP >10.0 mg/L as the threshold for excluding patients with acute infections.

The consent procedure was approved by the Institutional Review Board of Beijing Hui-long-guan Hospital. We recruited patients if they or their parent/legal guardian/next of kin provided written informed consent.

### 2.2 Clinical Assessment

Patients' symptoms were assessed using the Positive and Negative Syndrome Scale (PANSS) [24]. Before the study, all interviewers received extensive training on administering the PANSS scale. After training, the intraclass correlation coefficients (ICCs) for the PANSS total score exceeded 0.8.

### 2.3 Blood Measurement

Blood samples were extracted on the second day of hospitalization for the first-episode psychosis of patients. The blood sample was taken from each patient between 7:00 AM and 8:00 AM. WBC counts were calculated by the SYSMEX XN-3000 assembly line in the hospital. The measurements of WBC were carried out in strict accordance with the operation manual.

### 2.4 Statistical Analysis

Differences in the demographic data and WBC counts between patients and healthy controls, as well as between males and females, were analyzed using univariate analysis of variance (ANOVA) for the continuous variables or chi-square for the categorical variables.

To examine whether the sex effect existed only in patients, we explored the interaction effect of diagnostic group (patients *vs.* healthy controls) and sex (male *vs.* female) on NLR values or NEU counts using a 2 × 2 ANOVA. The two-way ANOVA was also used to test the interaction effect of sex (male *vs*. female) and NLR or NEU (high- *vs*. low subgroup) on clinical symptoms after dividing all patients into two subgroups based on the NEU or NLR median value.

Correlations of demographic and clinical data with WBC counts or NLR were analyzed using Pearson’s correlation analysis in patients and controls. Multiple linear regression analyses were performed to quantify the amount of variance in clinical data explained by WBC counts and NLR in male and female patients, after controlling for age, years of education, and onset age.

All analyses used SPSS version 20.0. The significance threshold was set at *p* < 0.05. Multiple comparisons were adjusted by Bonferroni’s correction. G*Power 3.1.9.2 program was applied to calculate the power.

## 3 RESULTS

NLR values and NEU counts were higher in FES patients compared to healthy controls (NLR: *p* = 0.003; NEU counts: *p* = 0.045). After Bonferroni's corrections, the difference in NLR remained significant (Bonferroni corrected *p <* 0.05). There was no significant difference in LYM counts between patients and controls (*p*>0.05).

Of all the 97 patients, 55 (56.7%) were male, and 42 (43.3%) were female. Two-way ANOVA showed that there was a significant main effect of the diagnostic group (F = 9.3, *p* = 0.003) and a significant interaction of sex × group (F = 4.0, *p* = 0.048) on NLR values, as well as an interaction effect of sex × group (F = 3.8, *p* = 0.05) on NEU counts. Post hoc comparisons showed that NEU counts were higher in males than females only in the patient group (F = 5.85, *p* = 0.02) but not in the control group (Table **1**). In addition, male patients had higher NLR values than female patients (F = 4.79, *p* = 0.03), but not in the control group. After controlling for age and education years, the differences in NEU counts and NLR values between male and female patients remained significant (all *p <* 0.05). However, there was no significant sex difference in LYM counts in patients (F = 1.13, *p* = 0.29).

The two-way ANOVA was performed to test the interaction between sex and WBC count on clinical symptoms. We found a significant effect of sex × NLR subgroup on positive symptoms (F = 6.8, *p* = 0.01). In addition, there was a significant sex × NEU subgroup on negative symptoms (F = 4.1, *p* = 0.046).

To examine the association between WBC counts and clinical symptoms in patients, Pearson’s correlation analyses were also used to determine the associations between WBC and symptoms in males and females. As shown in Table **2**, in males, there were significant associations between NEU counts and negative symptoms, general psychopathology, and PANSS total scores (all *p <* 0.05). In female patients, NLR values significantly correlated with positive symptoms, general psychopathology, and PANSS total score (all *p <* 0.05).

We further quantified the amount of variance in PANSS scores explained by WBC counts using linear regression analysis in both female and male patients. The results revealed that in female patients, NLR values were independently associated with the positive symptoms after controlling for age, years of education, and onset age (β = 0.413, t = 2.948, *p* = 0.006). In male patients, NEU counts were independently associated with negative symptoms after controlling for these confounders (β = 0.568, t = 2.328, *p* = 0.024).

## 4 DISCUSSION

This study showed that NEU counts and NLR values were higher in male patients with schizophrenia than in female patients, while there was no significant sex difference in healthy controls. Furthermore, significant correlations of NLR and NEU counts with clinical symptoms in patients showed sex differences. The findings of our study provide further evidence for the significant sex differences in schizophrenia.

We found sex differences in NLR values in patients with schizophrenia but not in healthy controls. Male patients had higher NLR values than female patients. Our findings are consistent with previous studies [25, 26]. However, some studies on NLR in schizophrenia have reported opposite findings between males and females, and even that there was no sex difference in schizophrenia [27, 28]. It is speculated that several factors, including interethnic differences related to the immune system, differences in WBC measurements, differences in sampling (serum or plasma), patients at different stages of the disease (chronic *versus* acute or active stage *versus* remission), exposure to different antipsychotics, and the dosage and duration of antipsychotic medication, may account for the discrepancy. To minimize the influence of confounding factors on NLR values and to make it a reliable biomarker in clinical practice, more studies focusing on investigating different cutoff values for different ethnic groups and different disease stages of schizophrenia are necessary. In addition, future studies should adopt standardized protocols to ensure comparability of measurements across laboratories.

In addition, we found higher NEU counts in male patients than in female patients, suggesting a sex-based disparity in immune system activation. It remains unclear whether sex differences in NEU and NLR are caused by biological or secondary environmental factors [29, 30]. NEU is the first line of immune defense, exhibiting phagocytic and apoptotic action through the secretion of a variety of inflammatory mediators, especially cytokines [15]. According to previous findings, higher NLR was positively correlated with pro-inflammatory cytokines in patients, such as tumor necrosis factor (TNF-α), interleukin (IL)-6, and IL-8 in patients [31-33]. Increased NEU counts in male patients indicate that low-grade inflammation was more pronounced in males than in females [34]. Increased NEU counts lead to elevated levels of inflammatory cytokines, and inflammation may further induce cellular dysfunction and oxidative stress. These processes are thought to be associated with the pathophysiology of schizophrenia and other mental health disorders.

Notably, we further found that NLR values were associated with positive symptoms in female patients, and NEU count was associated with negative symptoms in male patients, suggesting sex differences in the involvement of immune cells in schizophrenia. Immune cells and related cytokines have been reported to be closely associated with neurodevelopment in early adulthood. NLR is known to be a useful biomarker for detecting inflammatory responses, reflecting the intensity of stress and systemic inflammation, as well as the downstream cytokine cascade significantly correlated with schizophrenia [35]. In particular, NLR reflects the balance between the innate immune response and the adaptive immune response. Previous studies have reported significant differences in immune responses between males and females, with females having superior humoral and cell-mediated immunity than males in the general population [36]. Inflammatory cytokines mediate alterations in neurotransmission that are closely related to the pathological mechanisms of schizophrenia. Recent evidence also supports that sex hormones, such as estrogen, progesterone, and testosterone, have a direct effect on immune cell function and inflammatory capacity [37-40]. It is reported that women have a reduced prevalence of bacterial, viral, and parasitic infections and a stronger immune response to certain vaccines. However, our study showed no significant sex differences in NLR and NEU values in healthy controls.

This study had several limitations. First, many other inflammatory biomarkers during immune activation and inflammatory response were not assessed, which may lead to some bias. In particular, all patients with schizophrenia were experiencing their first episode of acute psychotic symptoms, and some factors, such as depression, anxiety, stress, and sleep disturbance, may have triggered an exaggerated immune response. Second, this was a cross-sectional study, and we were unable to draw firm conclusions about the relationship between NEU and NLR values and the pathophysiology of schizophrenia from the design of this study. Third, due to the difficulties in recruiting FES patients, the sample size of this study was small, and further studies with a large sample size were needed to validate our preliminary findings. Fourth, there is evidence that NEUs are very sensitive to cortisol levels. Mature NEUs stay in the bone marrow and are quickly released in blood flow when cortisol rises. However, we did not measure blood cortisol levels, and future research was needed to investigate the association between them.

## CONCLUSION

The current study reveals that NLR values and NEU counts were higher in male patients compared to female FES patients suffering from schizophrenia. Increased NEU counts correlated with symptoms only in male patients, whereas increased NLR correlated with symptoms only in female patients. Our findings add to the current understanding of sex differences in WBC counts and NLR in schizophrenia. However, these results may not be generalized to patients with chronic schizophrenia. Further studies are warranted to better understand whether the increase in NLR and NEU counts is altered in symptomatic remission with a longitudinal or prospective design.

## Figures and Tables

**Table 1:** Demographic and clinical data of cases and controls.

**Variable**	**Cases (n = 97)**				**Healthy Controls (n = 65)**			
	**Males n = 55**	**Females n = 42**	**F**	***P***	**Males n = 42**	**Females n = 23**	**F**	***P***
Age (yrs)	24.0 ± 6.4	24.2 ± 7.6	0.02	0.89	24.9 ± 6.0	24.5 ± 6.4	0.07	0.79
Education(yrs)	8.1 ± 2.8	8.0 ± 2.8	0.14	0.91	12.3 ± 3.3	10.0 ± 3.2	7.50	0.01
Onset age (yrs)	23.3 ± 6.4	23.6 ± 7.7	0.04	0.84	-	**-**	-	-
NLR	2.8 ± 1.6	2.1 ± 1.2	4.79	0.03	1.9 ± 0.8	1.8 ± 1.0	0.02	0.89
NEU	4.8 ± 1.8	4.0 ± 1.5	5.85	0.02	4.0 ± 1.2	3.9 ± 1.6	0.09	0.77
LYM	2.0 ± 0.8	2.1 ± 0.9	1.13	0.29	2.3 ± 0.7	2.3 ± 0.8	0.01	0.92
PANSS scale	-	-	-	-	-	-	-	-
P	19.0 ± 4.5	19.6 ± 5.3	0.34	0.56	-	-	-	-
N	18.4 ± 7.2	18.5 ± 6.9	0.01	0.95	-	-	-	-
G	32.6 ± 6.2	32.6 ± 8.2	<0.001	0.99	-	-	-	-
Total score	70.0 ± 12.8	70.0 ± 14.6	<0.001	0.99	-	-	-	-

**Note:** *yrs:* years.

Results are presented as a mean+/-SD for indicated number of subjects.

**Table 2:** Correlations between NLR and clinical symptoms in patients.

**-**	**-**	***P* Subscore**	**N Subscore**	**G Subscore**	**PANSS Total Score**
***Male Patients (n = 55)***					
NLR	r	0.09	0.19	0.13	0.21
	p	0.50	0.16	0.33	0.13
LYM	r	0.07	-0.02	0.13	0.08
	p	0.62	0.91	0.33	0.56
NEU	r	0.14	0.44	0.42	0.50
	p	0.31	**0.001**	**0.002**	**<0.001**
***Female Patients (n = 42)***					
NLR	r	0.36	0.13	0.45	0.41
	p	**0.02**	0.40	**0.003**	**0.006**
LYM	r	-0.11	-0.12	-0.38	-0.27
	p	0.49	0.45	**0.01**	0.08
NEU	r	0.24	0.15	0.11	0.23
	p	0.13	0.36	0.47	0.14

## Data Availability

The data and supportive information are available within the article.

## LIST OF ABBREVIATIONS

NEU: Neutrophil
NLR: Neutrophil-to-lymphocyte Ratio
PANSS: Positive and Negative Syndrome Scale
WBC: White Blood Cells
